# Distinct and Coordinated Regulation of Small Non-coding RNAs by E2f1 and p53 During *Drosophila* Development and in Response to DNA Damage

**DOI:** 10.3389/fcell.2021.695311

**Published:** 2021-07-22

**Authors:** Dong Li, Ying Ge, Ze Zhao, Rui Zhu, Xiang Wang, Xiaolin Bi

**Affiliations:** ^1^School of Medicine, Nantong University, Nantong, China; ^2^Institute of Cancer Stem Cell, Dalian Medical University, Dalian, China; ^3^College of Basic Medical Sciences, Dalian Medical University, Dalian, China

**Keywords:** miRNA, piRNA, E2f1, p53, *Drosophila melanogaster*, DNA damage response

## Abstract

Small non-coding RNAs (ncRNAs), including microRNAs (miRNAs) and PIWI-interacting RNAs (piRNAs), play a pivotal role in biological processes. A comprehensive quantitative reference of small ncRNAs expression during development and in DNA damage response (DDR) would significantly advance our understanding of their roles. In this study, we systemically analyzed the expression profile of miRNAs and piRNAs in *wild-type* flies, *e2f1* mutant, *p53* mutant and *e2f1 p53* double mutant during development and after X-ray irradiation. By using small RNA sequencing and bioinformatic analysis, we found that both miRNAs and piRNAs were expressed in a dynamic mode and formed 4 distinct clusters during development. Notably, the expression pattern of miRNAs and piRNAs was changed in *e2f1* mutant at multiple developmental stages, while retained in *p53* mutant, indicating a critical role of E2f1 played in mediating small ncRNAs expression. Moreover, we identified differentially expressed (DE) small ncRNAs in *e2f1* mutant and *p53* mutant after X-ray irradiation. Furthermore, we mapped the binding motif of E2f1 and p53 around the small ncRNAs. Our data suggested that E2f1 and p53 work differently yet coordinately to regulate small ncRNAs expression, and E2f1 may play a major role to regulate miRNAs during development and after X-ray irradiation. Collectively, our results provide comprehensive characterization of small ncRNAs, as well as the regulatory roles of E2f1 and p53 in small ncRNAs expression, during development and in DNA damage response, which reveal new insights into the small ncRNAs biology.

## Introduction

Small non-coding RNAs (ncRNAs) are short (∼ 20–30 nt) endogenous RNAs, which play diversified regulatory roles at physiological conditions and during pathological processes. Based on biogenesis and modes of regulation to targets, small ncRNAs are categorized into several classes, including microRNA (miRNA), small interfering RNA (siRNA), and PIWI-interacting RNA (piRNA) (Esteller, 2011; Ghildiyal and Zamore, 2009). Although with great difference, many small ncRNAs in different classes work competitively or collaboratively to regulate genes expression during development and in response to stress.

miRNAs are the most extensively studied small ncRNAs. They are 20–22 nucleotides long, and play pivotal roles in essentially all developmental processes by post-transcriptional regulation of genes expression, and their dysregulation are closely connected with various diseases (Alvarez-Garcia and Miska, 2005; Bartel, 2018). Considering the crucial roles that miRNAs play, miRNAs expression has been profiled in various cell lines and tissues, at different developmental stages, and in diverse organisms (Aravin et al., 2003; Biemar et al., 2005; de Rie et al., 2017; Ehrenreich and Purugganan, 2008; Graveley et al., 2011; He et al., 2017; Landgraf et al., 2007; Rahmanian et al., 2019; Wienholds et al., 2005). In *Drosophil*a, miRNAs functions were studied in many aspects, such as germline, central nervous system, immunity, behavior etc (Carthew et al., 2017), and systemic analysis of miRNAs were performed in embryos (Biemar et al., 2005), testes (Aravin et al., 2003), and different developmental stages (Aravin et al., 2003; Berezikov et al., 2011).

As a closely related yet completely different small ncRNA class, piRNAs are longer small RNAs (∼ 25–30 nt), and their sequence are highly diverse with little in common except the first nucleotide at the 5’ end (Ghildiyal and Zamore, 2009; Huang et al., 2017). piRNAs were identified in the *Drosophila* germline to repress transposon activity to preserve the genome integrity (Rojas-Rios and Simonelig, 2018). In addition, piRNAs have functions independent of transposon element and play roles to regulate genes expression, mRNA localization, stem cell biology etc (Rojas-Rios and Simonelig, 2018). Although the piRNA is a larger group of small ncRNAs, and there are millions of piRNA reads in genome, the global expression profiling of piRNAs during development is still missing, thus impeding the functional and mechanistic exploration of piRNAs in *Drosophila*.

In addition to regulating genes expression during development, miRNAs and piRNAs play an essential role in stress response to internal and environmental stimuli, such as starvation, oxidative stress, and genotoxic stress (Babar et al., 2008; Belicard et al., 2018; Belicard et al., 2018). Accumulating evidences suggest that small ncRNAs play a vital role in DNA repair and maintenance of genome integrity, among which the miRNA is the most well-studied (d’Adda di Fagagna, 2014). miRNAs work at two levels in DNA damage response (DDR). The expression level of miRNAs can be modulated by genotoxic stress. After DNA damage, miRNAs are transcriptionally regulated by transcriptional factors such as p53 (Liao et al., 2014), E2f1 (Knoll et al., 2013), Myc (Psathas and Thomas-Tikhonenko, 2014) etc. miRNAs can also regulate expression of critical DDR factors such as double-strand break (DSB) sensors, mediators, and effectors (Chowdhury et al., 2013; Gandellini et al., 2014). The transcription factor p53, known as the “guardian of the genome,” is a key regulator of DDR. Upon DNA damage, p53 is activated and performs multitasks to protect genome integrity. Activated p53 can arrest cell cycle transiently or permanently, mediate apoptosis, and is involved into a variety of DNA repair machineries (Williams and Schumacher, 2016). E2f1 is another transcription factor that plays critical role in DDR. In response to DNA damage, E2f1 is phosphorylated by ataxia telangiectasia mutated (ATM), ATM and Rad3-related (ATR) kinases or checkpoint kinase 2 (Chk2) and stabilized, which in turn activates the transcription of its downstream apoptotic genes, and promotes the recruitment or retention of DDR factors at the DSB (Biswas and Johnson, 2012; Poppy Roworth et al., 2015). The p53 and E2f1 transcription factors have extensive crosstalk to perform cellular functions. They share the common upstream regulators ATM, Chk1/Chk2 at DDR. Meanwhile, E2f1 can regulate p53 pathway both positively and negatively through regulating downstream genes, which form a feed-forward loop to regulate apoptosis (Polager and Ginsberg, 2009).

It is known that miRNAs are important players in the p53 signaling pathway. p53 can regulate transcription and biogenesis of miRNAs by affecting the RNA-induced silencing complex (RISC) complex functions. miRNAs can also regulate p53 and its working partners to regulate p53-dependent cell cycle arrest and apoptosis. Studies on p53-mediated miRNAs and the feedback regulation of p53 by miRNAs were extensively performed in the last few decades (Liao et al., 2014), while the study about E2f1-miRNAs network is relatively less. E2f1 can regulate several families or clusters of miRNAs, such as miR-15 family, miR-34 family, miR-17-92 cluster, miR-106b-25 cluster etc. Most of these miRNAs form a feedback loop with E2f1 and mediate classical E2f1 functions to regulate cell cycle progression or apoptosis (Knoll et al., 2013; Poppy Roworth et al., 2015). Besides miRNAs, piRNAs are also implicated in the DNA damage response. Mutations in the piRNA pathway can lead to a significant over-expression of retrotransposons and a high level of DNA damage (Khurana et al., 2010). Till now, genome-wide *in vivo* analysis of p53 or E2f1 regulated small ncRNAs during development or in response to ionizing radiation is still lacking, and the coordination of E2f1 and p53 to regulate small ncRNAs is still poorly understood.

Although a simple metazoan, the *Drosophila melanogaster* has been used as a model organism to elucidate basic biological processes and intrinsic mechanisms for over a century, and most of the identified signaling pathways are highly conserved in *Drosophila*. Knowledge from *Drosophila* studies is being widely used to answer questions about human diseases (Bier, 2005), especially the inter-crosstalk among key signaling pathways. In this study, we describe a comprehensive atlas of the small ncRNAs expression during development and after ionizing radiation in *wild-type* flies, *e2f1* mutant, *p53* mutant, and *e2f1 p53* double mutant. Both miRNAs and piRNAs exhibited similar and stage-specific expression pattern during development, and E2f1 plays a prominent role in regulating miRNAs and piRNAs expression during development. Moreover, small ncRNAs regulated by p53 after X-ray irradiation were mostly different in embryos and third instar larvae. E2f1 also played an important role to regulate miRNAs and piRNAs in response to DNA damage, and E2f1 and p53 work coordinately to regulate miRNAs and piRNAs after DNA damage. Furthermore, we mapped the binding motif of E2f1 and p53 toward small ncRNAs.

## Results

### miRNAs Show a Dynamic Mode of Expression During Development

**FIGURE 1:**
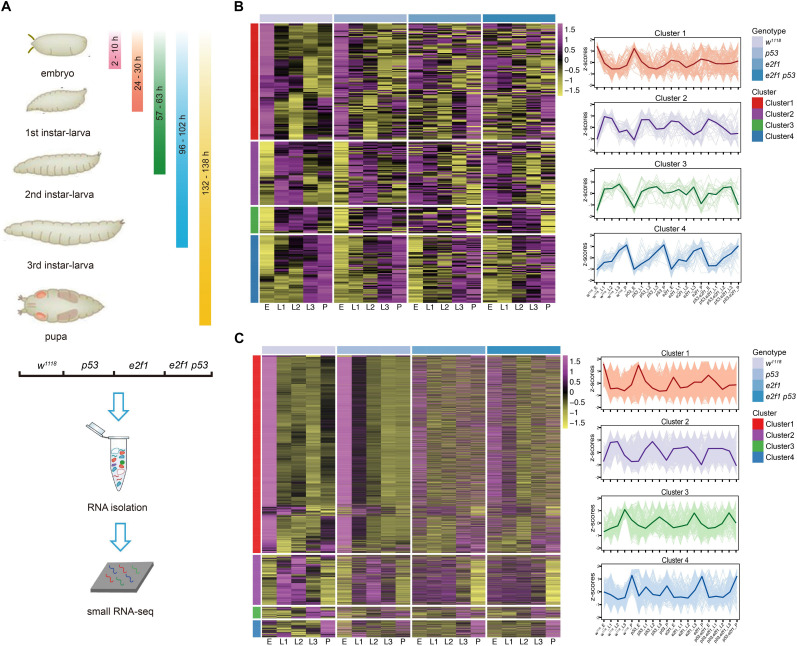
Clustering of *Drosophila* miRNAs and piRNAs during development. **(A)** Schematic illustration of experimental design and time-points for samples collection. Heatmap of the normalized expression level of miRNAs **(B)** and piRNAs **(C)** in the 4 clusters in *w*^1118^, *p53* mutant, *e2f1* mutant and *e2f1 p53* double mutant.

To systematically investigate the expression dynamics of small ncRNAs across *Drosophila* developmental stages, we performed small RNA-Seq analysis using total RNA isolated from whole-animal samples at five different developmental stages, embryo, L1 larva, L2 larva, L3 larva and pupa, from *wild-type* flies, *e2f1 mutant*, *p53 mutant*, and *e2f1 p53* double mutant (Figure 1A). We generated a dataset comprising a total of 75 small RNA sequencing libraries, and there are on average 32 million reads per library. For the miRNA analysis, mirPRo pipeline was used to map raw reads to *Drosophila melanogaster* genome (Ensembl, BDGP6.28), annotate known miRNAs, and identify novel miRNAs. Based on the miRbase (V21 and V22.1), 454 known mature miRNAs were identified in all samples, and 248 miRNAs of them belong to 109 miRNA families (Supplementary Table 1). In addition, 1,596 novel mature miRNAs were predicted based on mirDeep2 and RNAfold algorithms integrated in mirPRo (Supplementary Table 1). For the piRNA analysis, we obtained expression values by mapping reads to *Drosophila* piRNA sequences (piRNAdb.dme.v176.fa) using the Salmon aligner. A total of 20,462 piRNAs were identified in all samples (Supplementary Table 2).

To characterize the genome-wide small RNAs involved in *Drosophila* development, stage-specific expression analysis was carried out to reveal the temporal expression pattern of small ncRNAs. Our data showed that miRNAs exhibited a dynamic mode of expression during development. miRNAs can be grouped into four distinct clusters based on expression profiles. The cluster 1 miRNAs were highly expressed in embryos in *wild-type* flies and *p53* mutant, yet they were not expressed differentially at other developmental stages in *wild-type* flies and *p53* mutant and at all developmental stages in *e2f1* mutant and *e2f1 p53* double mutant, suggesting the important roles of these miRNAs at embryonic stage. The cluster 2 miRNAs showed high expression at both L1 and L2 stages in all genotypes, and low expression at embryonic stage in *wild-type* flies and *p53* mutant, while no differential expression in *e2f1* mutant and *e2f1 p53* double mutant at embryonic stage. The cluster 3 miRNAs showed high expression at L3 stage in all genotypes, and low expression at embryonic stage in *wild-type* flies and *p53* mutant. The cluster 4 miRNAs had the lowest expression level at embryonic stage, increased their expression gradually during development and reached the highest level at pupae stage in *wild-type* flies and all three mutated flies (Figure 1B and Supplementary Table 3). We also analyzed the predicted novel miRNAs, and found a similar expression pattern as annotated miRNAs (Supplementary Figure 1 and Supplementary Table 3). Therefore, miRNAs exhibit a dynamic mode of expression during development. While the expression mode of miRNAs in *wild-type* flies and *p53* mutant was highly resembled, it was changed greatly at some developmental stages in *e2f1* mutant and *e2f1 p53* double mutant, especially at embryonic stage, indicating an important role of E2f1 in regulating biogenesis of miRNAs during early development.

### piRNAs Show a Similar Expression Pattern as miRNAs During Development

Surprisingly, the expression mode of piRNAs was quite similar, with slight difference, as miRNAs during development. Based on expression mode, piRNAs can also be grouped into four clusters. The cluster 1 piRNAs showed an almost identical mode of expression as the cluster 1 miRNAs in *wild-type* flies and all mutated flies. The cluster 2 piRNAs showed high expression at L1 and L2 stages in all genotypes, and low expression at embryonic stage in *wild-type* flies and *p53* mutant, which were similar as that of miRNAs, they were also expressed at low lever at pupae stage in all genotypes. The cluster 3 piRNAs showed high expression at L3 stage in all genotypes, and low expression at embryonic stage in *wild-type* flies, but not in *p53* mutant which is different when compared with that of miRNAs. The cluster 4 piRNAs showed high expression at pupae stage in *wild-type* flies, *e2f1* mutant and *e2f1 p53* double mutant, but not in *p53* mutant, and they did not show gradually increased expression trend either (Figure 1C and Supplementary Table 4). The expression pattern of piRNAs was more complexed in different genetic backgrounds, which might be caused by the large number of piRNAs existed. The similar expression mode of miRNAs and piRNAs suggested that they are regulated in a coordinated way during development, and E2f1 and p53 may perform similar roles in regulating miRNAs and piRNAs during *Drosophila* development.

### Functional Signaling Pathways Enrichment Analysis

**FIGURE 2:**
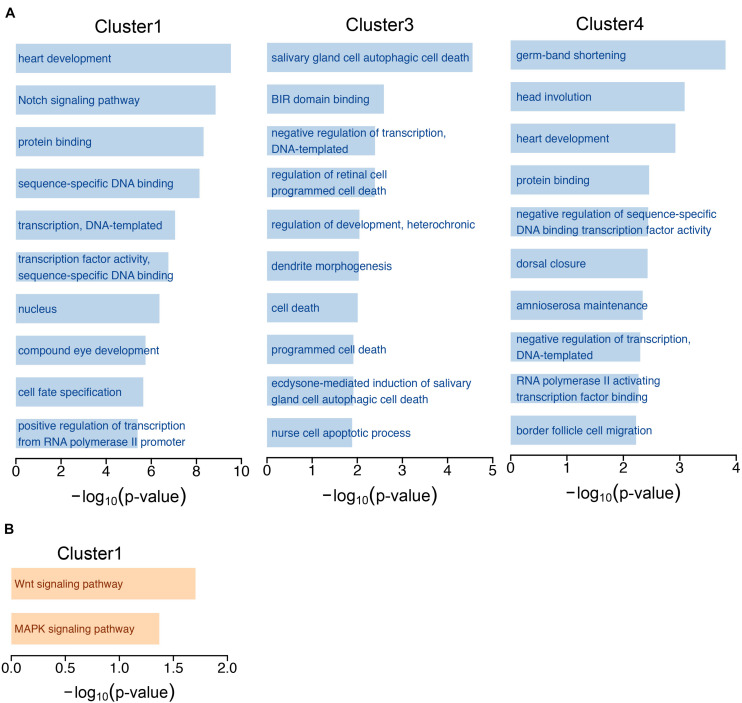
GO annotation and KEGG pathway analysis. **(A)** The top 10 GO terms of experimentally validated targeted genes of miRNAs in the 4 clusters. **(B)** Pathway analysis of targeted genes of cluster 1 miRNAs by KEGG.

Next, we performed GO and KEGG analysis based on the experimentally validated targeted genes of miRNAs in 4 clusters during development. The top 10 significantly enriched signaling pathways were identified and shown in cluster 1, cluster 3 and cluster 4 miRNAs, while we did not identify enriched signaling pathways from targeted genes of miRNAs in cluster 2 (Figure 2A and Supplementary Table 5). The GO functional annotations of cluster 1 miRNAs were highly enriched in development and transcription, which is reasonable as cluster 1 miRNAs were highly expressed at embryonic stage in *wild-type* flies. The cluster 3 miRNAs were more specific to cell death, miRNAs in cluster 3 showed high expression at L3 stage, and mild high level at L1 and L2 stages (Figure 1B). During larval stage, the body mass is increased by ∼ 200-fold (Church and Robertson, 1966), the cluster 3 miRNAs might play critical roles to regulate *Drosophila* development at larval stage by inhibiting cell death. The cluster 4 miRNAs were prone to development. In addition, the KEGG pathway analysis suggested that targeted genes of the cluster 1 miRNAs were enriched in the Wnt signaling pathway and MAPK signaling pathway (Figure 2B and Supplementary Table 5), indicating miRNAs regulated Wnt and MAPK signaling pathways are important at embryonic stage.

### E2f1 Plays a Prominent Role to Regulate Small ncRNAs During Development

To explore the roles that E2f1 and p53 perform in regulating small ncRNAs during development, we further compared the expression profiles of small ncRNAs among all genotypes. Loss of p53 did not change the expression pattern of miRNAs at distinct development stages when compared with that of *wild*-*type* flies (Figure 1B), or piRNAs except in cluster 3 and cluster 4 (Figure 1C), which is reasonable, as p53 is not essential for development, and *p53* knockout flies show little phenotype. In comparison, the expression pattern of miRNAs and piRNAs were changed significantly in multiple clusters at multiple developmental stages in *e2f1* mutant and *e2f1 p53* double mutant. High level expression of miRNAs and piRNAs at embryonic stage in cluster 1 in *wild*-*type* flies and *p53* mutant was almost diminished in *e2f1* and *e2f1 p53* mutants, and miRNAs in cluster 2 and cluster 3 and piRNAs in cluster 2 at embryonic stage showed low level expression in *wild*-*type* flies and *p53* mutant but were not expressed differentially or slightly increased in *e2f1* and *e2f1 p53* mutants (Figure 1C). These data suggested that E2f1 works as a critical regulator of miRNAs and piRNAs expression during early development. The expression pattern of miRNAs and piRNAs in *e2f1 p53* double mutant is similar as that of *e2f1* mutant, which further confirms that E2f1, not p53, plays a more important role for the dynamic expression of miRNAs and piRNAs during *Drosophila* development.

**FIGURE 3:**
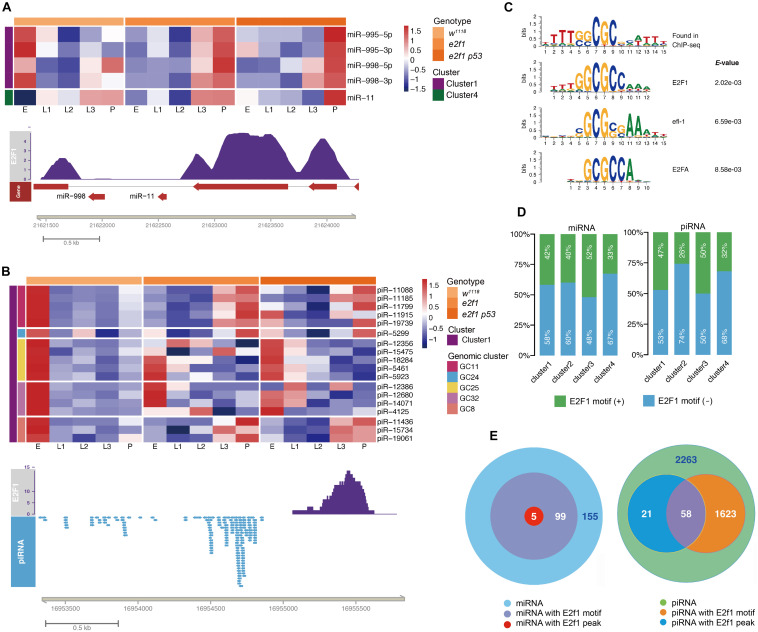
E2f1 regulated small ncRNAs during development. Illustrations of miRNAs **(A)** and piRNA **(B)** with E2f1 binding-peak. **(C)** E2f1 consensus motif derived from MEME de novo motif analysis. **(D)** Proportion of miRNAs or piRNAs in 4 clusters containing E2f1 binding-motif. **(E)** Venn diagram showing the overlap of E2f1 binding-peak and E2f1 binding-motif around miRNAs or piRNAs gene locus.

Next, we sought to investigate the E2f1 binding motif and identify small ncRNAs that could be regulated directly by E2f1. To determine the *in vivo* binding motif of E2f1, we utilized the public E2f1 ChIP-seq data (ERR268501), and identified 5 miRNAs and 79 piRNAs in clusters 1 - 4 had V5 tagged E2f ChIP-seq peaks (Figures 3A,B and Supplementary Tables 6,7). It is noteworthy that we mapped the binding motif around *miR-11* and *miR-998*, the two miRNAs in the first intron of *e2f1* gene and suppress E2f1-dependent cell death (Truscott et al., 2011; Truscott et al., 2014), these miRNAs had reversed expression pattern in *e2f1* mutant and *e2f1 p53* double mutant when compared with that of *wild-type* flies (Figure 3A). We further defined the new motif centrally enriched in the top 1000 peaks in *Drosophila*, in which the consensus sequence is similar as the E2f1 binding-motif in human, *C*. *elegans*, and Arabidopsis (Figure 3C). We localized this motif by scanning the promoter (∼1 kb), gene body and downstream region of the gene locus (∼1 kb). We identified 104 miRNAs (Supplementary Table 5), which takes 40.1% (104/259) of miRNAs in clusters 1–4, and 1,681 piRNAs (Supplementary Table 7), which takes 42.4% (1,681/3,965) of piRNAs in cluster 1–4, with E2f1 motif, respectively. Moreover, E2f1 binding-motif was identified in 42% of miRNAs and 47% of piRNAs in cluster 1, 40% of miRNAs and 26% of piRNAs in cluster 2, and 52% of miRNAs and 50% piRNAs in cluster 3, while only 33% of miRNAs and 32% piRNAs in cluster 4 (Figures 3D,E). The proportion of miRNAs with E2f1 binding-motif in cluster 4 is lower than that in clusters 1–3, which is consistent with the data that E2f1 is dispensable for the dynamic expression of small miRNAs in cluster 4 (Figure 1C). While the proportion of piRNAs with E2f1 binding-motif in cluster 4 is lower than that in cluster 1 and cluster 3, higher than that in cluster 2. These data provided further evidences that E2f1 regulates the dynamic expression of small ncRNAs during development.

### p53 Regulates Small ncRNAs Differently in Response to Radiation at Different Developmental Stages

**FIGURE 4:**
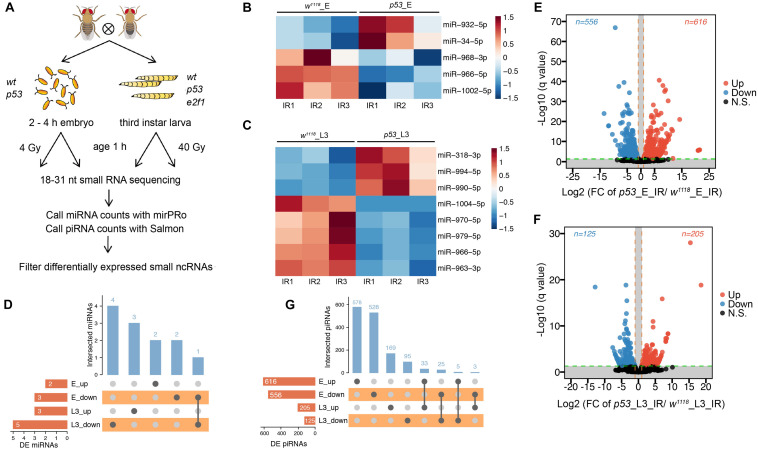
DE miRNAs and piRNAs after X-ray irradiation in *p53* mutant. **(A)** Schematic illustration of experimental design and time-points for samples collection. Heatmap of DE miRNAs after DNA damage in *p53* mutant embryos **(B)** and L3 **(C)**. **(D)** UpSet plot depicting the number of DE miRNAs in response to DNA damage in *p53* mutant embryos and L3. Volcano plots of piRNAs differentially expressed between *wild-type* flies and *p53* mutant embryos **(E)** or L3 **(F)** after X-ray irradiation. **(G)** UpSet plot depicting the number of DE piRNAs in response to DNA damage in *p53* mutant embryos and L3.

To investigate small ncRNAs in response to ionizing radiation (IR), actively crawling third instar larvae (L3) of *wild*-*type* flies, *p53* mutant and *e2f1* mutant were treated with 40 Gy, and embryos of *wild*-*type* flies and *p53* mutant were treated with 4 Gy, respectively (Figure 4A). The total RNA was prepared at 1 h after X-ray irradiation, and then RNA-seq was performed. The ncRNA gene expressed with significant difference (fold change ≥2, adjusted *P* < 0.05) between the treated group and control group in 3 replicates was regarded as differentially expressed (DE). After X-ray irradiation, we identified 5 DE miRNAs in *p53* mutated embryos, including *miR-932* and *miR-34*, which were up-regulated, and *miR-968*, *miR-966* and *miR-1002*, which were down-regulated (Figure 4B and Supplementary Table 8). The expression profiles of these 5 DE miRNAs were further validated by RT-qPCR (Supplementary Figure 2). And 8 miRNAs in L3, including *miR-994*, *miR-318* and *miR-990*, which were up-regulated, and *miR-970*, *miR-966*, *miR-979*, *miR-963*, and *miR-1004*, which were down-regulated (Figure 4C and Supplementary Table 8). Majority of DE miRNAs in *p53* mutated embryos and L3 were different. To be specific, 4 of 5 DE miRNAs were specific in embryos, and 7 of 8 DE miRNAs were specific in L3 (Figure 4D). Only one miRNA, *miR-966*, was down-regulated in both *p53* mutated embryos and L3. We also identified 39 upregulated and 26 downregulated novel miRNAs in *p53* mutated embryos, as well as 10 upregulated and 5 downregulated novel miRNAs in *p53* mutated L3 (Supplementary Figure 3 and Supplementary Table 8). Similar as annotated miRNAs, majority of DE novel miRNAs in *p53* mutated embryos and L3 were different.

Furthermore, 1,172 DE piRNAs were identified in *p53* mutated embryos after X-ray irradiation, including 616 up-regulated and 556 down-regulated, and 330 DE piRNAs in L3, including 205 up-regulated and 125 down-regulated (Figures 4E,F and Supplementary Table 9). Among which, only 58 piRNAs, including 33 up-regulated and 25 down-regulated, were differentially expressed in both *p53* mutated embryos and L3 (Figure 4G). These data indicated that small ncRNAs regulated by p53 in response to radiation were altered at different developmental stages, and p53 might perform its functions through different set of small ncRNAs in response to DNA damage during development.

### E2f1 Plays a More Important Role in Regulating miRNAs in Response to Ionizing Radiation

**FIGURE 5:**
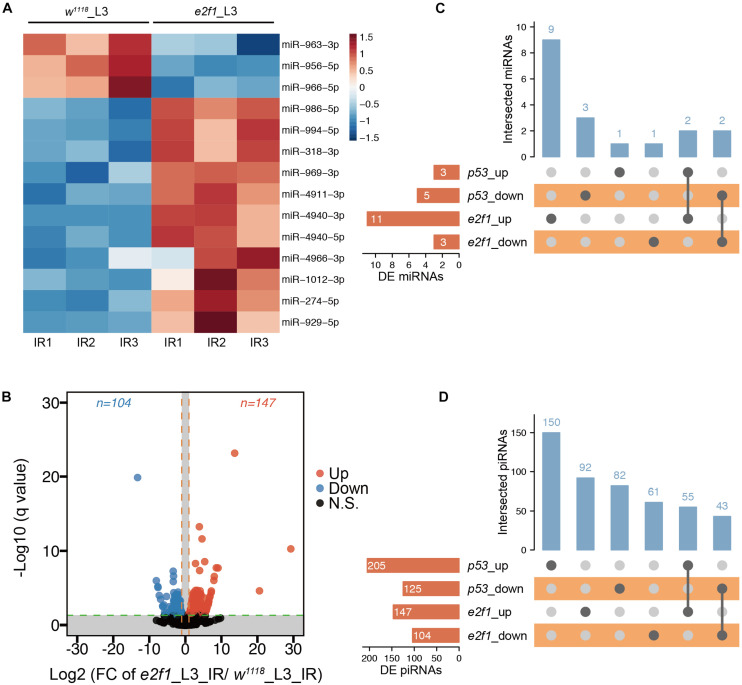
DE miRNAs and piRNAs after X-ray irradiation in *e2f1* mutant. **(A)** Heatmap of the DE miRNAs after DNA damage in *e2f1* mutant L3. **(B)** UpSet plot depicting the number of DE miRNAs in response to DNA damage in *p53* and *e2f1* mutant L3. **(C)** Volcano plots of piRNAs differentially expressed between *wild-type* flies and *e2f1* mutant L3 after X-ray irradiation. **(D)** UpSet plot depicting the number of DE piRNAs in response to DNA damage in *p53* and *e2f1* mutant L3.

We further compared the RNA-seq data between the *e2f1* mutant and *wild-type* flies. We identified 14 DE miRNAs, including 11 up-regulated and 3 down-regulated, in *e2f1* mutated L3 after X-ray irradiation (Figure 5A and Supplementary Table 10). Moreover, 251 DE piRNAs, including 147 up-regulated and 104 down-regulated, were identified in *e2f1* mutated L3 (Figure 5B and Supplementary Table 11). We also identified 6 upregulated and 4 downregulated novel miRNAs in *e2f1* mutated L3 (Supplementary Figure 4 and Supplementary Table 10). The number of DE miRNAs in *e2f1* mutant is a lot more than that in *p53* mutant in L3 after X-ray irradiation, indicating that E2f1 plays a more important role to regulate miRNAs in response to radiation at the third instar larval stage.

We further compared DE miRNAs and DE piRNAs between *e2f1* mutant and *p53* mutant in L3. We found that 4 miRNAs were expressed in a similar trend in both *e2f1* and *p53* mutants, including up-regulated *miR-994* and *miR-318* and down-regulated *miR-966* and *miR-963* (Figure 5C), implicating these miRNAs function as common downstream targets of both E2f1 and p53 in response to DNA damage. Notably, *miR-966* was down-regulated in *p53* embryos, *p53* L3 and *e2f1* L3 mutants after X-ray irradiation. Moreover, 55 piRNAs were up-regulated and 43 piRNAs were down-regulated in both *e2f1* and *p53* mutants (Figure 5D and Supplementary Table 12).

### Expression of miRNA Clusters and Families During Development and After X-Ray Irradiation

**FIGURE 6:**
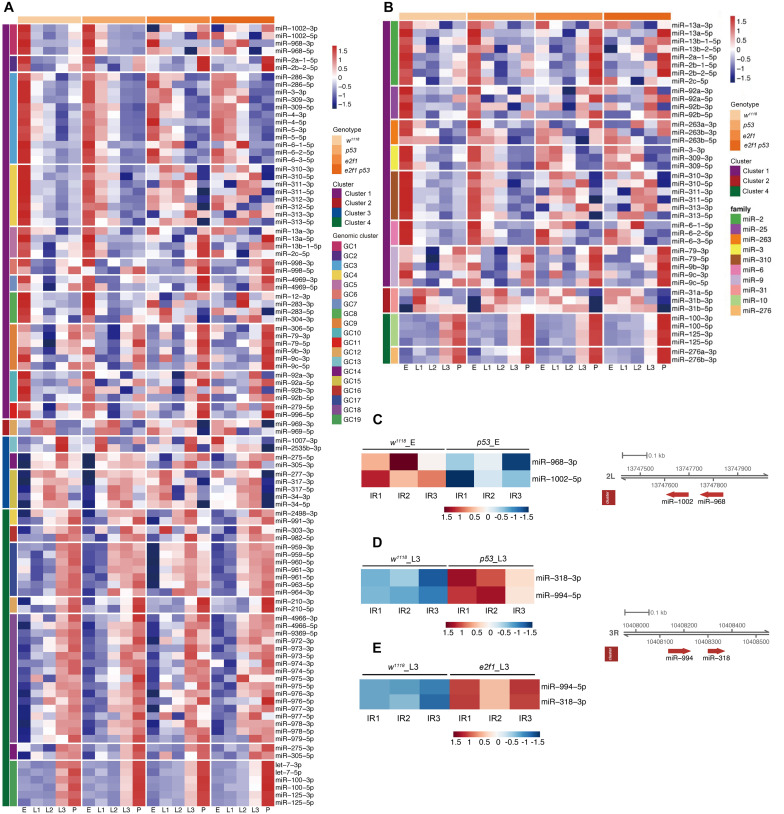
Expression of miRNA genomic clusters and families during development and after X-ray irradiation. **(A)** Heatmap of miRNA genomic clusters during development. **(B)** Heatmap of miRNA families during development. **(C)** Heatmap of DE miRNA clusters after X-ray irradiation in *p53* mutant embryos. **(D)** Heatmap of DE miRNA clusters after X-ray irradiation in *p53* mutant L3. **(E)** Heatmap of DE miRNA clusters after X-ray irradiation in *e2f1* mutant L3.

miRNAs in gene cluster or family may have functional relationships via coregulating biological processes. Many miRNAs locate closely at the chromosomal locus and form a cluster. The clustered miRNAs may have common cis-regulatory elements, and work coordinately to regulate multiple biological processes in a polycistron (Zhang et al., 2009). The miRNAs that derived from identical ancestor in the phylogenetic tree are usually grouped into a family, and family members share similar seed sequence and perform similar functions. To investigate how miRNAs in *Drosophila* form clusters and play roles during development and after DNA damage, we defined a cluster as chromosome distance at 10 kb window between two miRNAs in the same orientation, and performed the miRNA cluster finding in the *Drosophila* genome BDGP 6.28. We identified 19 miRNA clusters including 99 miRNAs and 10 miRNA families including 41 miRNAs, respectively, in which miRNAs exhibited coordinately expression pattern during development (Figures 6A,B and Supplementary Table 13). Among these clusters, the *miR-2a-1*/*2a-2*/*2b-2* and *miR-13a*/*13b-1*/*13b-2* clusters were expressed abundantly in embryos, while *miR-79*/*9c* cluster exhibited high expression in embryos and pupae, which is consistent with previously described expression pattern of these miRNAs during development (Sempere et al., 2003; Ruby et al., 2007). These three miRNA clusters belong to two miRNA families. The *miR-2a-1*/*2a-2*/*2b-2* and *miR-13a*/*13b-1*/*13b-2* clusters belong to the *miR-2* family, which have been shown to play important roles in regulating cell death and morphogenesis (Chandra et al., 2017). The *miR-79/9c* cluster belongs to the *miR-9* family, which is involved into the regulation of germ cell development (Kugler et al., 2013). The *miR-2a-1*/*2a-2*/*2b-2* and *miR-13a*/*13b-1*/*13b-2* clusters exhibited not only internally consistent expression pattern, but also showed highly correlated expression, implying that members of the *miR-2* family work coordinately and are regulated coordinately at transcriptional level during early development.

We further analyzed the miRNA clusters after X-ray irradiation. Among the DE miRNAs in *p53* mutant, *miR-968* and *miR-1002* in *miR-968-1002* cluster were down-regulated in *p53* embryos, while *miR-318* and *miR-994* in *miR-318-994* cluster is up-regulated in *p53* L3 (Figures 6C,D). *miR-318* is involved in chorion-containing eggshell pattern formation and eggshell chorion gene amplification (Ge et al., 2015), while the function of *miR-994* is unknown. Notably, expression of *miR-318-994* cluster is also up-regulated in *e2f1* L3 (Figure 6E), indicating that *miR-318-994* cluster works as common down-stream target of both E2f1 and p53 in response to DNA damage at third instar larval stage.

### Expression of piRNA Clusters During Development and After X-Ray Irradiation

**FIGURE 7:**
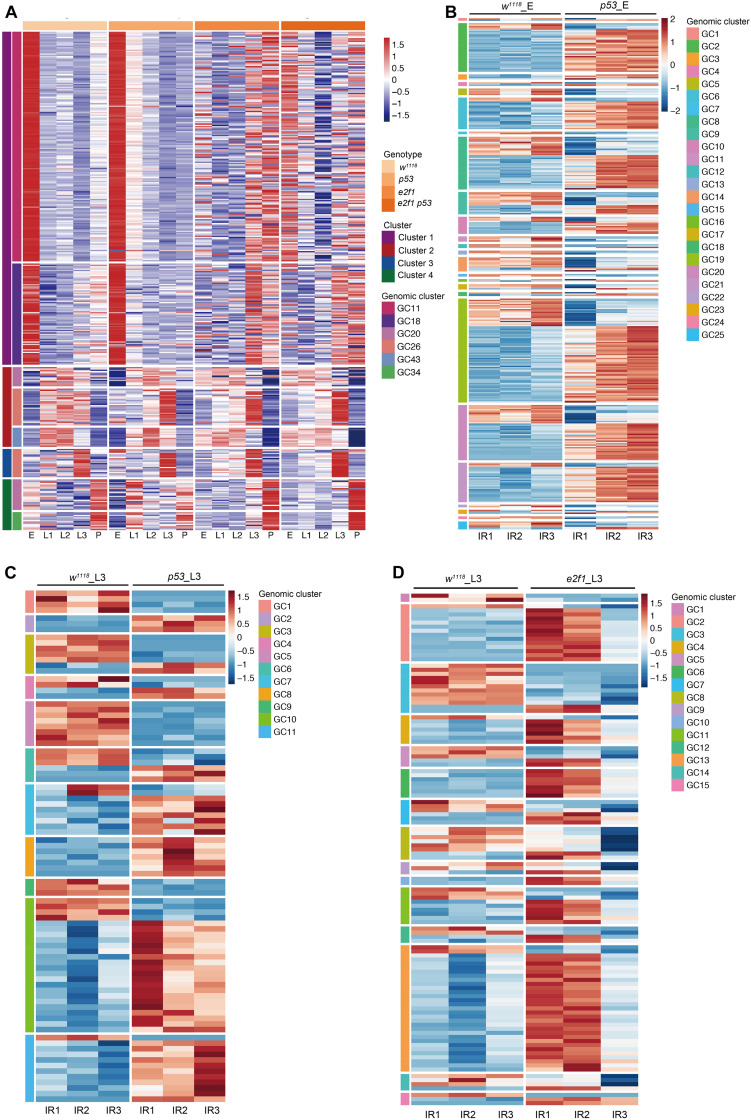
Expression of piRNA clusters during development and after X-ray irradiation. **(A)** Representative illustration of heatmap of piRNA genomic clusters during development. **(B)** Heatmap of DE piRNA clusters after X-ray irradiation in *p53* mutant embryos. **(C)** Heatmap of DE piRNA clusters after X-ray irradiation in *p53* mutant L3. **(D)** Heatmap of DE piRNA clusters after X-ray irradiation in *e2f1* mutant L3.

Most piRNAs are derived from genomic piRNA clusters. We next investigate how piRNAs in *Drosophila* form clusters and play roles during development and after DNA damage. We identified 43 piRNA clusters, consisting of 1,275 piRNAs, showing coordinately expression pattern during development (Figure 7A and Supplementary Table 14).

We further analyzed the piRNA clusters after X-ray irradiation. Among the DE piRNAs in *p53* mutant, 343 DE piRNAs formed 25 genomic clusters in *p53* embryos, and 87 DE piRNAs formed 11 genomic clusters in *p53* L3 (Figures 7B,C and Supplementary Table 15). Moreover, in *e2f1* L3, 117 DE piRNAs formed 15 genomic clusters (Figure 7D and Supplementary Table 16).

### Characterization of p53 Binding Motif in the Small ncRNA Gene Region

The p53 family consists of p53, p63 and p73 in mammals, p53 is the only member in *Drosophila* and performs canonical apoptotic function at DNA damage response and a context-dependent cell cycle arrest (Mollereau and Ma, 2014). Although a total of 3,661 target genes of p53 were identified by meta-analysis approaches, only 346 genes were verified in individual studies (Fischer, 2017). We therefore sought to determine the small ncRNAs that might be directly regulated by p53 in *Drosophila*.

**FIGURE 8:**
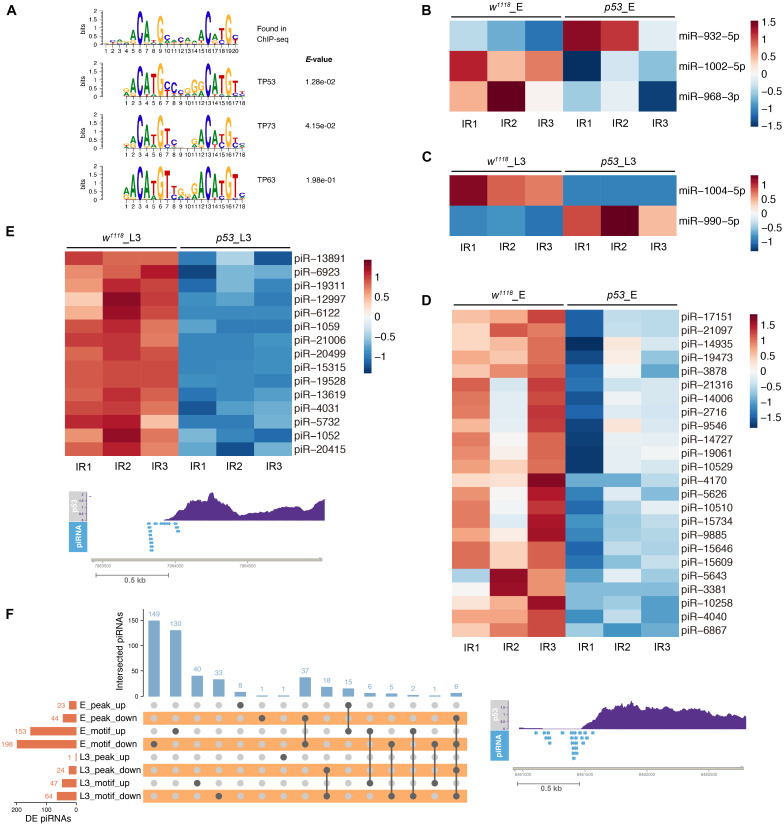
p53 regulated small ncRNAs in response to X-ray irradiation. **(A)** p53 consensus motif derived from MEME de novo motif analysis. **(B)** Heatmap of DE miRNAs in *p53* mutant embryos containing p53 binding-motif. **(C)** Heatmap of DE miRNAs in *p53* mutant L3 containing p53 binding-motif. **(D)** Representative illustration of DE piRNAs in *p53* mutant embryos **(D)** and L3 **(E)** containing p53 binding-peak. **(F)** UpSet plot depicting the number of DE piRNAs with p53 binding-peak and p53 binding-motif.

Firstly, we detected p53 targeted miRNAs by referencing modERN project eGFP-p53 ChIP-seq data ENCSR808XNJ in 0–24 h embryos. We analyzed the p53 binding motif around *Drosophila* miRNAs based on the p53 ChIP-seq data. The canonical human p53, p63 and p73 motifs were found enriched in the top 1000 peaks (Figure 8A). We scanned the p53, p63 and p73 motifs in promoter, gene body and 3’ downstream region of DE miRNA in *p53* mutant after X-ray irradiation. Among the 5 DE miRNAs in *p53* mutated embryos, 3 miRNAs, including *miR-932*, *miR-968*, *miR-1002*, have the p53/63/73 consensus binding-motif. And 2 out of 8 DE miRNAs in *p53* mutated L3, including *miR-990* and *miR-1004*, have the p53 motif (Figures 8B,C). Notably, 5 of 13 DE miRNAs found in *p53* after DNA damage have the p53/63/73 consensus binding-motif, which makes a strong suggestion that these miRNAs are regulated directly by p53.

Next, we analyzed the p53 binding peak and motif around the DE piRNAs. Among the 1,172 DE piRNAs identified in *p53* mutated embryos after X-ray irradiation, 67 piRNAs have p53 binding peaks and 351 piRNAs have the p53 binding motif (Figures 8D,F and Supplementary Table 17). In 330 DE piRNAs identified in *p53* mutated L3, 25 piRNAs have p53 binding peaks and 111 piRNAs have the p53 binding motif (Figures 8E,F and Supplementary Table 17).

### Characterization of E2f1 Binding Motif in the Small ncRNA Gene Region After X-Ray Irradiation

**FIGURE 9:**
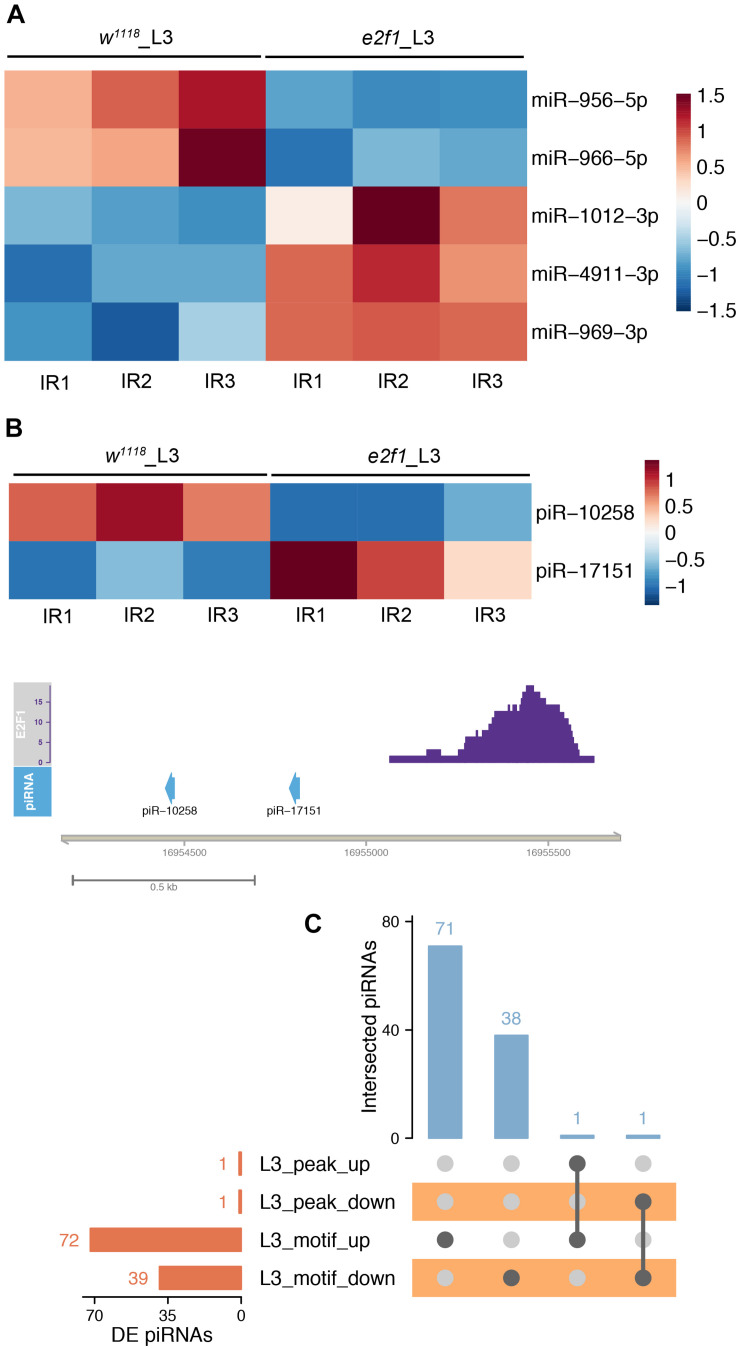
E2f1 regulated small ncRNAs in response to X-ray irradiation. **(A)** Heatmap of DE miRNAs in *e2f1* mutant L3 containing E2f1 binding-motif. **(B)** Illustration of DE piRNAs in *e2f1* mutant L3 containing E2f1 binding-peak. **(C)** UpSet plot depicting the number of DE piRNAs with E2f1 binding-peak and E2f1 binding-motif.

We further investigated the E2f1 binding peaks and motif to identify the DE small ncRNAs that could be regulated directly by E2f1 after X-ray irradiation. Among 14 DE miRNAs in *e2f1* mutated L3, 5 DE miRNAs, including *miR-956*, *miR-966*, *miR-969, miR-1012*, and *miR-4911*, were found containing E2f1 binding motif (Figure 9A). Two DE piRNAs have E2f1 binding peak and 111 DE piRNAs have E2f1 binding motif (Figures 9B,C and Supplementary Table 18).

### Irradiation Sensitivity of miRNA Alleles

We next investigated the radiosensitivity the DE miRNA mutated flies. Among the 22 DE miRNAs after X-ray irradiation, we tested 7 DE miRNAs that have publicly available knock-out (KO) (Chen et al., 2014) mutants. The actively crawling late third instar larvae of the DE miRNA mutants were collected, and treated with X-ray irradiation at dosage of 0, 10, 20, 30, and 40 Gy, respectively. The irradiated larvae were let grow, the survival adults were counted, and the survival percentage was calculated.

**FIGURE 10:**
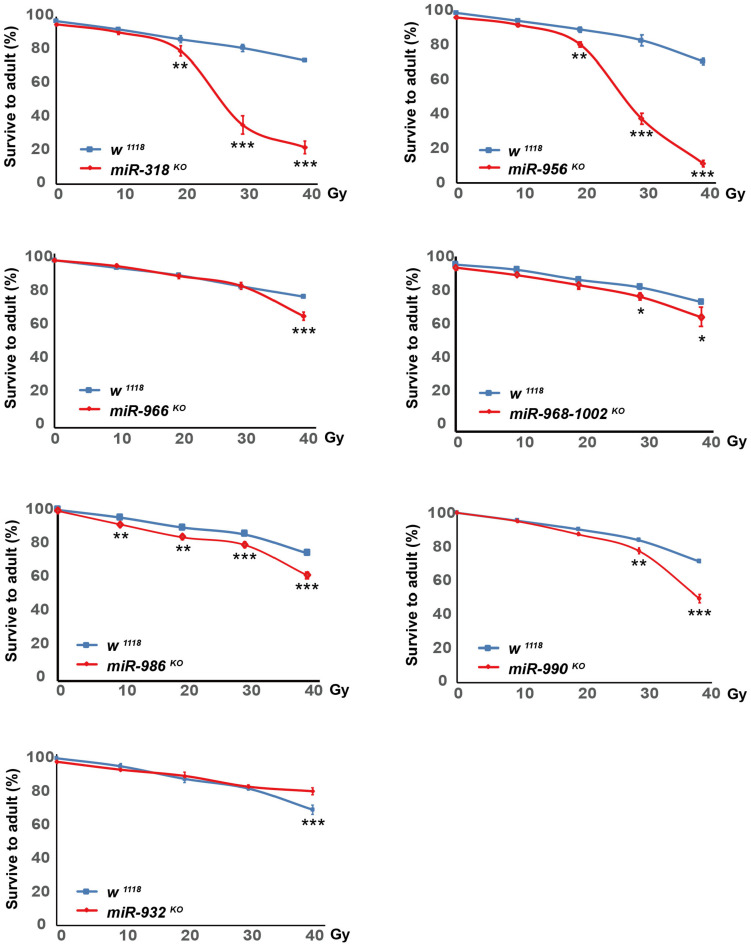
Sensitivity of DE miRNAs mutants to X-ray irradiation. Late third instar larvae of *w^1118^, miR-318 ^*KO*^*, *miR-932 ^*KO*^*, *miR-956 ^*KO*^*, *miR-966 ^*KO*^*, *miR-968-1002 ^*KO*^*, *miR-986 ^*KO*^* and *miR-990 ^*KO*^* flies were irradiated with dosage of 0, 10, 20, 30, and 40 Gy, respectively. Survival adults were counted, and survival percentage was calculated as number of viable adult flies divided by number of irradiated larvae. At least 100 larvae were treated at each dosage for each genotype, and three independent experiments were performed. *P ≤ 0.05, **P ≤ 0.01, ***P ≤ 0.001; error bars indicate SEM.

We found that *miR-318 ^*KO*^*, *miR-956 ^*KO*^*, *miR-968-1002 ^*KO*^*, *miR-986 ^*KO*^*, and *miR-990 ^*KO*^* flies were more sensitive to multiple dosage of X-ray irradiation when compared with that of *wild-type* flies. The *miR-966 ^*KO*^* flies were sensitive to 40 Gy X-ray irradiation, while *miR-932 ^*KO*^* flies were resistant to 40 Gy X-ray irradiation (Figure 10). These results provided further evidences that these DE miRNAs played important roles in mediating DNA damage response.

## Discussion

Small ncRNA plays a vital role in development and stress responses by orchestrating key biological processes and fine tuning the protein-coding genes expression. Elucidating the expression dynamics of small ncRNAs is important to understand the gene regulation networks and physiological functions in biological processes. Although studies have shown the developmental stage specificity and stress responsiveness of small ncRNAs in multiple model organisms, a comprehensive *in vivo* study of the dynamics of small ncRNAs during development and in response to DNA damage under p53 or E2f1, the two key regulators of DNA damage response, mutant background were still lacking. Our study fills this knowledge gap. In this study, we provide a comprehensive resource of small ncRNAs expression dynamics across *Drosophila* developmental stages and after DNA damage in *wild-type*, *p53* mutated, *e2f1* mutated and *e2f1 p53* double mutated flies.

Based on the analysis, we found that miRNAs and piRNAs were grouped into 4 distinct clusters and expressed dynamically during development. Interestingly, piRNAs exhibited similar expression mode with that of miRNAs during development, suggesting a potential regulatory mechanism to coordinate miRNAs and piRNAs expression. We also identified 19 miRNA and 43 piRNA genomic clusters that were co-regulated during development, which is consistent with the idea that miRNAs and piRNAs are often forming clusters and expressed coordinately. The dynamic expression pattern of miRNAs and piRNAs at embryonic stage, but not at later developmental stages, was largely disrupted in *e2f1* mutant, which strongly suggests that E2f1 is one of the key regulators of miRNAs and piRNAs biogenesis during early development. As a key regulator of cell proliferation, E2f1 is essential for development. Our results demonstrated that several miRNAs involved in negative regulation of cell death, such as miR-2 and miR-6 family members, were highly expressed at embryonic stage in *wild-type* flies, but downregulated in *e2f1* mutant. E2f1 binding-motif was also identified around multiple miRNAs and piRNAs loci, which further indicates the direct regulation of miRNAs and piRNAs by E2f1. On the other hand, the expression mode of miRNAs in *p53* mutant was comparable to that of *wild-type* flies, suggesting a minor role of p53 plays in regulating miRNAs expression during development. However, expression of piRNAs in two clusters at L3 larva or pupae stage was changed in *p53* mutant, indicating p53 may play a role to regulate piRNAs at later stage of development. Thus, our data reveal the distinct regulation of small ncRNAs expression by E2f1 and p53 during development.

p53 and E2f1 are two critical mediators of DNA damage response. Our analysis revealed several p53- or E2f1-dependent miRNAs and piRNAs in response to radiation. We identified 5 DE miRNAs in *p53* mutated embryos and 8 DE miRNAs in *p53* mutated L3 after X-ray irradiation, the majority of which were different, suggesting p53 may regulate distinct miRNAs in response to DNA damage at different developmental stage. On the other hand, we identified more DE miRNAs in *e2f1* mutated L3 after X-ray irradiation, indicating a more important role of E2f1 in mediating miRNAs expression after X-ray irradiation compared with that of p53. More importantly, many DE miRNAs (4/8) and DE piRNAs (143/360) in the *p53* mutant were also differentially expressed in the *e2f1* mutant after X-ray irradiation. These data suggest the coordinate regulation of miRNAs and piRNAs by E2f1 and p53 after DNA damage.

To understand biological roles that DE miRNAs might play after DNA damage, we investigated the radiosensitivity of 7 DE miRNA mutants. Six DE miRNA mutants showed increased sensitivity to X-ray irradiation, while 1 DE miRNA mutant was resistant to 40 Gy X-ray irradiation. All of the DE miRNA mutants we tested showed changed sensitivity to X-ray irradiation, indicating critical roles of these DE miRNAs played in regulating DNA damage response.

Taken together, our study systematically analyzed the temporal expression pattern of small ncRNAs during development and in response to DNA damage under different genetic background, which provides a valuable resource for elucidating the role of small ncRNAs and highlighting key properties of small ncRNAs during *Drosophila* development and in response to DNA damage.

## Materials and Methods

### Fly Genetics

All flies were maintained at 25°C on standard corn meal unless specified. Fly lines used in this study were: *w*^ 1118^ (*wild-type*), *p53*^ 5*A*–1–4^ (*p53*, BL6815), *e2f1 ^07172^* (BL11717), *e2f1 ^*i2*^* (BL7274), *e2f1 ^07172^*/*e2f1 ^*i2*^* (*e2f1*), *e2f1 ^07172^ p53*/*e2f1 ^*i2*^ p53* (*e2f1 p53*), *tub*>Gal4, *act*>Gal4, UAS-dsRed, miRNA ^*KO*^ flies (Chen et al., 2014).

### Samples Collection for RNA-Seq

One embryos collection, 3 larvae collection, and 1 pupae collection were taken. Before each collection, at least two pre-lays were performed. Embryos were collected as 0–8 h eggs, allowed to grow another 2 h to obtain 2 - 10 h samples (E). Larvae and pupae were raised from 0 to 6 h eggs, and allowed to develop to the desired stage, and collected at 24–30 h as early first instar larvae (L1), 57–63 h as mid second instar larvae (L2), 96–102 h as mid-late third instar larvae (L3), and 132–138 h as pupae (P). The embryos were dechorionated and frozen in liquid nitrogen, larvae and pupae were frozen in liquid nitrogen.

### Ionizing Radiation

Embryos at 2–4 h of *wild-type* flies and *p53* mutant were irradiated with 4 Gy, and actively crawling third instar larvae of *wild-type* flies, *p53* mutant and *e2f1* mutant were irradiated with 40 Gy, at a dose rate of 340 cGy/min, with X-RAD 320 iX at 320 kV (Precision X-Ray, Inc., North Branford, CT, United States). Let the irradiated embryos and L3 larvae recovered for 1 h, and the control groups grow the same time. Embryos were washed into baskets using tap water, dechorionated in 50% bleach, washed thoroughly with tap water and dried with a tissue paper, and frozen immediately in liquid nitrogen for RNA preparation. The irradiated and recovered L3 larvae were frozen in liquid nitrogen for RNA preparation.

### RNA-Seq

Total RNA from embryos, L1, L2 and L3 larvae, and pupae was extracted using Trizol with DNase treatment at the Beijing Genomics Institute (BGI Co., Ltd., China). The integrity of extracted RNA was assessed by Agilent 2100 Bioanalyzer (Agilent Technologies, Palo Alto, CA, United States). The small RNA libraries were prepared with Illumina’s TruSeq Small RNA Sample Prep kit according to Illumina instructions. Single-end 50 bp sequencing was performed using Illumina HiSeq 2000 (IR treated samples) and BGISEQ-500 (samples at different developmental stages) platforms at the Beijing Genomics Institute (BGI Co., Ltd., China). The RNA-Seq experiment encompassed three biological replicates.

### RNA-Seq Data Analysis

For miRNAs analysis, raw sequencing data were aligned to the *Drosophila melanogaster* genome using mirPRo pipeline (Shi et al., 2015) with the following parameters: -m mature.fa -p hairpin.fa -f miFam.dat -s dme -a null -q 0 -r 1 –novel 1 –other hsa –other mmu -g Drosophila_melanogaster_genome.fa –gtf Drosophila_melanogaster.gtf –index Dme.novoindex -t 12. The sequence files (mature.fa, hairpin.fa) for known miRNAs were retrieved from the miRBase V22.1. The miFam.dat was downloaded from miRBase V21. The reference genome and gene annotation files (Drosophila_melanogaster_genome.fa, Drosophila_melanogaster.gtf) were downloaded from the Ensembl database (*Drosophila_melanogaster.BDGP6.28*, release-101). For piRNAs analysis, raw reads were mapped to known *Drosophila* piRNA sequences using the Salmon software with the following parameters: salmon quant -i piRNA_salmon_index –validateMappings -l A -p 10. The piRNA sequence file (piRNAdb.dme.v1_7_6.fa.gz) was downloaded from https://www.pirnadb.org. The identification of differentially expressed miRNAs or piRNAs was performed by DESeq2. A gene is considered differentially expressed if the absolute log2-fold change is ≥1 and the adjusted *p*-value is <0.05. Clustering was performed with differentially expressed genes during different development stages in *wild-type* flies using the built-in clustering algorithms of R package Pheatmap.

### Reverse Transcription-Quantitative PCR

Total RNA was reverse transcribed and the miRNA expression was quantified with a miRNA-specific qPCR protocol as previously described (Busk, 2014). Briefly, a poly(A) tail was added to the miRNA sequence with poly(A) polymerase (NEB, M0276S), and miRNA was reverse transcribed with an anchored poly(T) primer CAGGTCCAGTTTTTTTTTTTTTTTVN (Invitrogen, China), where V is A, C or G, and N is A, C, G or T. The miRNA transcripts were quantified by quantitative PCR (qPCR) with ABI StepOnePlus real-time PCR machine using the standard mode. Reactions were performed with Roche FastStart Universal SYBR Green Master (Rox) (Product no. 04913914001), and quantified with comparative Cq method using U6 as reference gene. Relative expression change was calculated from three independent experiments. The primers are listed in Supplementary Table 19.

### Enrichment Analysis for miRNA Targets

The experimentally validated *Drosophila* miRNA targets were downloaded from miRTarBase.^1^ The targeted genes of co-clustered mature miRNAs (cluster 1–4) at different stages were obtained by searching the downloaded files and subjected to gene ontology (GO) annotation and KEGG pathway enrichment analysis using The Database for Annotation, Visualization and Integrated Discovery (DAVID).^2^ The significantly involved GO terms and pathways (count cutoff 5 or 2, *p* < 0.05) were identified.

### miRNA Genomic Cluster Annotation

The genomic coordinates of all known primary miRNAs were retrieved from gene annotation files (dme.gff3) of miRBase V22.1. A miRNA cluster is defined as two miRNA genes with a maximum distance of 10 kb on the same chromosome and in the same strand. After determining all miRNA clusters annotation information, differentially expressed miRNAs were classified into clusters, with at least 2 mature miRNAs in each cluster.

### piRNA Genomic Cluster Annotation

The annotation information (list_cluster.v176.dm6) of all known piRNA clusters was downloaded from https://www.pirnadb.org. The differentially expressed piRNAs were classified into clusters, with at least 2 piRNAs in each cluster.

### Genome-Wide Distribution of E2f1 Binding Sites

The fastq file ERR268501 of E2f ChIP-seq (Orian et al., 2003) was downloaded from ftp://ftp.sra.ebi.ac.uk/. All subsequent data analysis steps were carried out using the WDL-based ENCODE Transcription Factor and Histone ChIP-Seq processing pipeline.^3^ The parameter for chip.peak_caller was set to macs2. MEME-ChIP (Machanick and Bailey, 2011) with default parameter was used to find motifs in up- and down-stream 200 bp fragments around all peaks. The newly discovered motif sequence most similar to known E2f1 motif was then used to scan *Drosophila* miRNAs or piRNAs gene body, upstream (∼1 kb) and downstream (∼1 kb) regions using MEME-FIMO.

### Genome-Wide Distribution of p53 Binding Sites

The fastq files of eGFP-p53 ChIP-seq datasets (ENCSR808XNJ) generated by Kevin White laboratory in modERN project were downloaded from https://www.encodeproject.org/. All subsequent data analysis steps were carried out using the WDL-based ENCODE Transcription Factor and Histone ChIP-Seq processing pipeline (see text footnote 3). MEME-ChIP with default parameter was used to locate motifs in up- and down-stream 300 bp fragments around top 1000 peaks. The newly discovered motif sequence most similar to known TP53 motif was then used to scan the *Drosophila* miRNAs or piRNAs gene body, upstream (∼1 kb) and downstream (∼1 kb) regions using MEME-FIMO.

### Viability Assay

Actively crawling third instar larvae were irradiated with the dosage of 0, 10, 20, 30, and 40 Gy, at a dose rate of 1.3 Gy/min in a X-Ray Biological Irradiator (X-RAD 320iX, Precision X-ray Inc), respectively. At least 100 larvae were irradiated for each dosage of each genotype, and experiments were repeated three times. Survival percentage was calculated as number of viable adults divided by number of irradiated third instar larvae.

## Data Availability Statement

The datasets presented in this study can be found in online repositories. The names of the repository/repositories and accession number(s) can be found below: https://www.ncbi.nlm.nih.gov/, PRJNA719871 and https://www.ncbi.nlm.nih.gov/, PRJNA719972.

## Author Contributions

XB designed the experiments. ZZ and RZ collected materials and performed the experiments. XW and YG performed bioinformatic analysis. DL, XW, and XB analyzed the data. DL and XB wrote the manuscript. All authors contributed to the article and approved the submitted version.

## Conflict of Interest

The authors declare that the research was conducted in the absence of any commercial or financial relationships that could be construed as a potential conflict of interest.

## Footnotes

1 http://mirtarbase.cuhk.edu.cn/

2 https://david.ncifcrf.gov/home.jsp

3 https://github.com/ENCODE-DCC/chip-seq-pipeline2
