# Supplementary material for: Distinct and Coordinated Regulation of Small Non-coding RNAs by E2f1 and p53 During Drosophila Development and in Response to DNA Damage
Source: Front Cell Dev Biol. 2021 Jul 22;9:695311. doi: 10.3389/fcell.2021.695311 (PMC8339594; doi:10.3389/fcell.2021.695311)
Supplement: Supplementary file 1 [file Data_Sheet_1.docx]

**Supporting information**

**
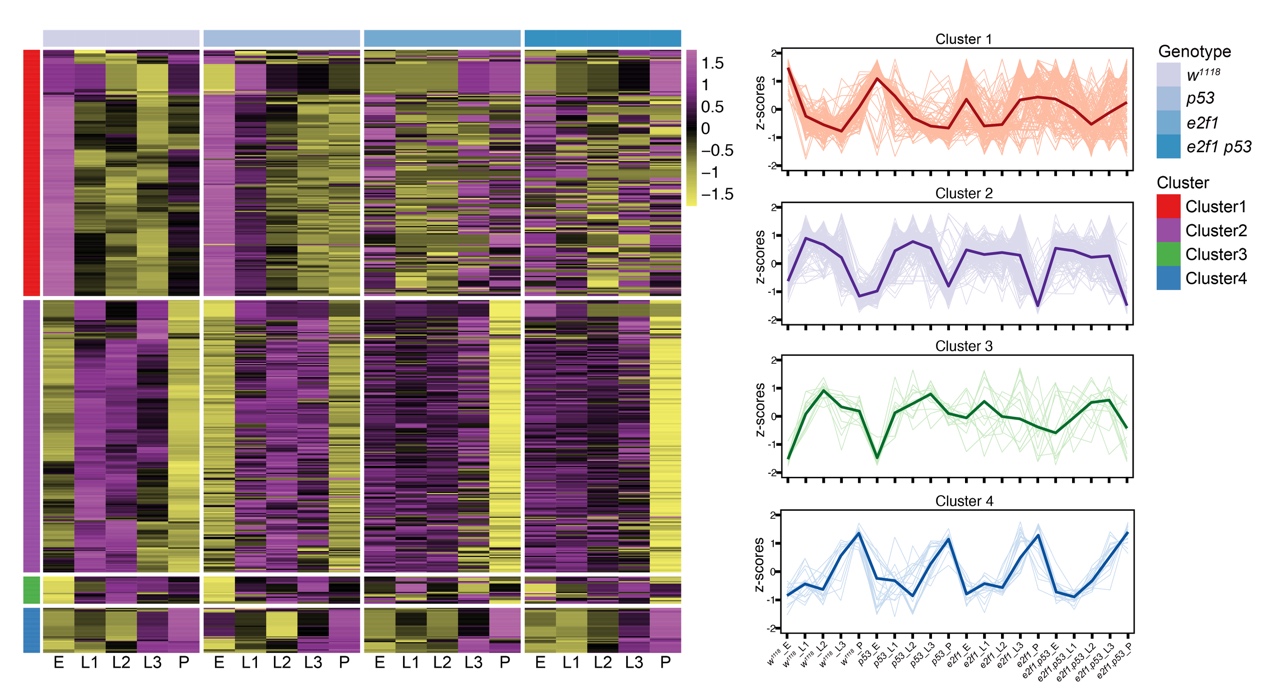
**

Figure S1. Clustering of *Drosophila* novel miRNAs during development. Heatmap of normalized expression levels of novel miRNAs in the 4 clusters in *w^1118^*, *p53* mutant, *e2f1* mutant and *e2f1 p53* double mutant.


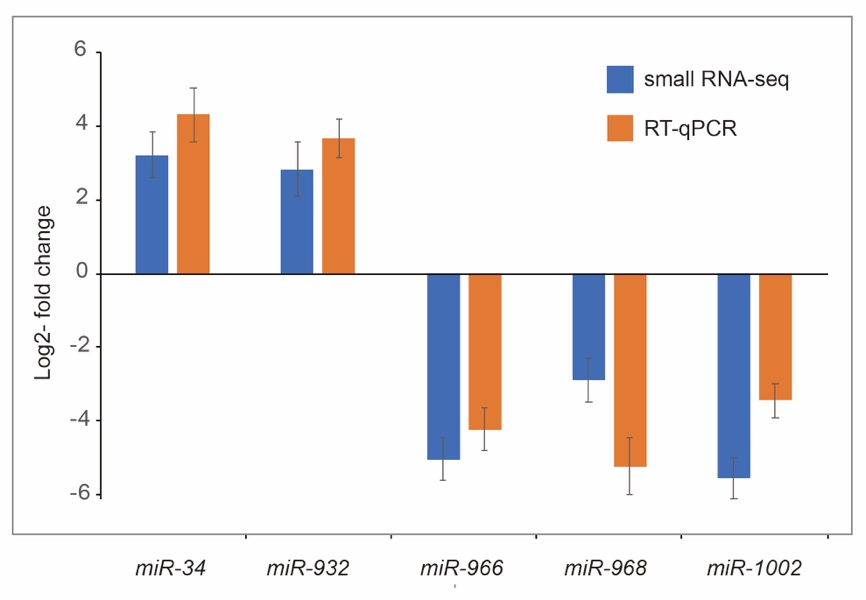


Figure S2. RT-qPCR validation of DE miRNAs identified by small RNA-seq. Validation of 5 DE miRNAs in *p53* mutated embryos after X-ray irradiation by RT-qPCR. The data shown are means ± SEM.


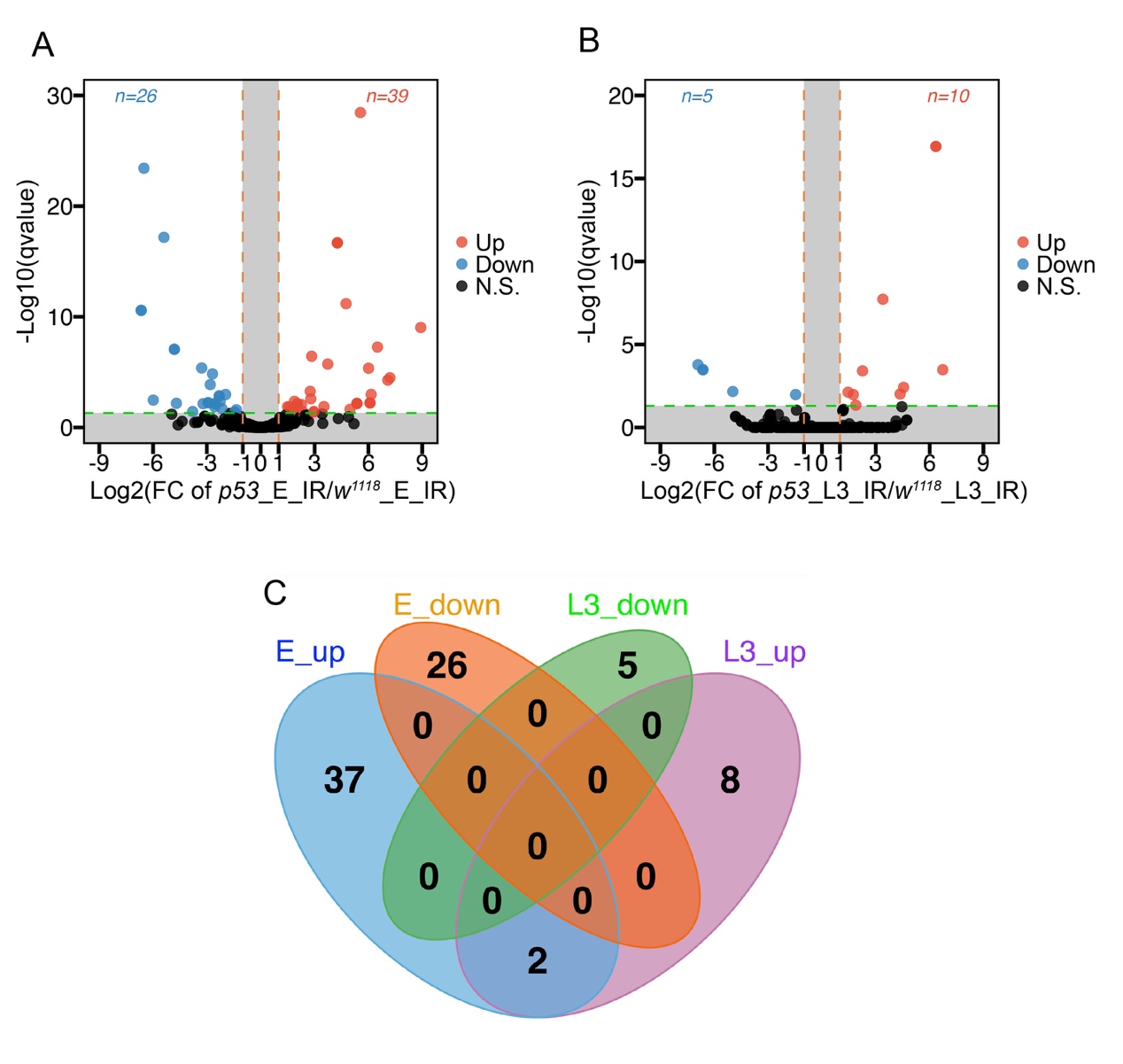


Figure S3. DE novel miRNAs after X-ray irradiation in *p53* mutant. (A) Volcano plots of piRNAs differentially expressed between *wild-type* flies and *p53* mutant embryos after X-ray irradiation. (B) Volcano plots of novel miRNAs differentially expressed between *wild-type* flies and *p53* mutant L3 after X-ray irradiation. (C) Venn diagram showing the overlap of DE novel miRNAs between *p53* mutant embryos and L3 after X-ray irradiation.


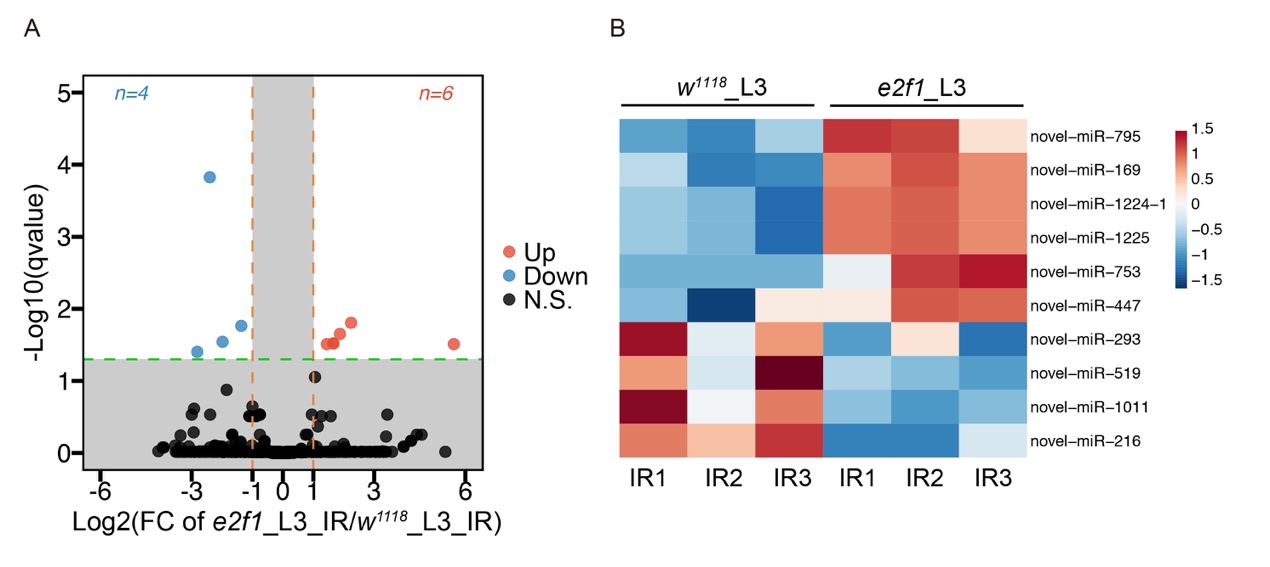


Figure S4. DE novel miRNAs after X-ray irradiation in *e2f1* mutant. (A) Volcano plots of novel miRNAs differentially expressed between *wild-type* flies and *e2f1* mutant L3 after X-ray irradiation. (B) Heatmap of novel miRNAs differentially expressed between *wild-type* flies and *e2f1* mutant L3 after X-ray irradiation.

Table S1. miRNA read count.

Table S2. piRNA read count.

Table S3. Clustering of miRNAs during development.

Table S4. Clustering of piRNAs during development.

Table S5. GO and KEGG analysis.

Table S6. miRNAs with E2f1 binding peak or motif in the 4 clusters during development.

Table S7. piRNAs with E2f1 binding peak or motif in the 4 clusters during development.

Table S8. DE miRNAs in response to DNA damage in *p53* mutant.

Table S9. DE piRNAs in response to DNA damage in *p53* mutant.

Table S10. DE miRNAs in response to DNA damage in *e2f1* mutant.

Table S11. DE piRNAs in response to DNA damage in *e2f1* mutant.

Table S12. DE piRNA between *p53* and *e2f1* mutant.

Table S13. miRNA families and genomic clusters during development.

Table S14. piRNA genomic clusters during development.

Table S15. DE piRNAs forming clusters after IR in *p53* mutant.

Table S16. DE piRNAs forming clusters after IR in *e2f1* mutant.

Table S17. DE piRNAs containg p53 binding peak and motif.

Table S18. DE piRNAs containg E2f1 binding peak and motif.

Table S19. Primers for RT-qPCR.
