# Supplementary material for: Distinct and Coordinated Regulation of Small Non-coding RNAs by E2f1 and p53 During Drosophila Development and in Response to DNA Damage
Source: Front Cell Dev Biol. 2021 Jul 22;9:695311. doi: 10.3389/fcell.2021.695311 (PMC8339594; doi:10.3389/fcell.2021.695311)
Supplement: Supplementary file 2 [file Data_Sheet.ZIP › Supplementary Tables/Table S19 Primers for RT-qPCR.docx]

Table S19 qPCR primers

| Name | Primer sequence (5'-3') |
| --- | --- |
| miR-1002-5p-F  miR-1002-R  miR-137-F  miR-137-R  miR-13a-3p-F  miR-13a-3p-R  miR-184-3p-F  miR-184-3p-R  miR-2b-1-3p-F  miR-2b-1-3p-R  miR-2b-2-F  miR-2b-2-R  miR-263b-F  miR-263b-R  miR-277-3p-F  miR-277-3p-R  miR-281-2-5p-F  miR-281-2-5p-R  miR-284-F  miR-284-R  miR-3-F  miR-3-R  miR-33-5p-F  miR-33-5p-R  miR-34-F  miR-34-R  miR-375-F  miR-375-R  miR-7-F  miR-7-R  miR-932-5p-F  miR-932-5p-R  miR-966-5p-F | GCAGTTAAGTAGTGGATACAAAG  GGTCCAGTTTTTTTTTTTTTTTAGTTAAG  GCAGTATTGCTTGAGAATACAC  GTCCAGTTTTTTTTTTTTTTTCTACG  CAGTATCACAGCCATTTTGATG  GGTCCAGTTTTTTTTTTTTTTTACTC  CAGTGGACGGAGAACTGA  CCAGTTTTTTTTTTTTTTTGCCCTT  AGTATCACAGCCAGCTTTG  GTCCAGTTTTTTTTTTTTTTTGCTC  CAGTTCTTCAAAGTGGTTGTG  GGTCCAGTTTTTTTTTTTTTTTCATTTC  GGTGGTTCTGCGGGT  TCCAGTTTTTTTTTTTTTTTGTTTTGG  GCAGTAAATGCACTATCTGGT  TCCAGTTTTTTTTTTTTTTTGTCGT  GCAGAAGAGAGCTATCCGT  GGTCCAGTTTTTTTTTTTTTTTACTGT  CCTGGAATTAAGTTGACTGTGT  TCCAGTTTTTTTTTTTTTTTGGCT  CACTGGGCAAAGTGTGT  GGTCCAGTTTTTTTTTTTTTTTGAGA  GGTGCATTGTAGTCGCA  GGTCCAGTTTTTTTTTTTTTTTGACA  GCAGTGTGGTTAGCTGGT  GTCCAGTTTTTTTTTTTTTTTCACAA  GCAGTTTGTTCGTTTGGCT  CAGGTCCAGTTTTTTTTTTTTTTTAAC  CGCAGCAATAAATCCCTTG  GTCCAGTTTTTTTTTTTTTTTAAGAAGAC  CGCAGTCAATTCCGTAGTG  CCAGTTTTTTTTTTTTTTTCTGCAATG  TGGGTTGTGGGCTGT |
| miR-1002-5p-R | GGTCCAGTTTTTTTTTTTTTTTTCGCCC |
| miR-34-5p-F | GCAGTGTGGTTAGCTGGT |
| miR-34-5p-R | GTCCAGTTTTTTTTTTTTTTTCACAA |
| miR-932-5p-F | CGCAGTCAATTCCGTAGTG |
| miR-932-5p-R | CCAGTTTTTTTTTTTTTTTCTGCAATG |
| miR-966-5p-F | TGGGTTGTGGGCTGT |
| miR-966-5p-R | GGTCCAGTTTTTTTTTTTTTTTCCA |
| miR-968-5p-F | CGCATAAGTAGTATCCATTAAAG |
| miR-968-5p-R | CCAGTTTTTTTTTTTTTTTCAACCC |
| U6-F | ACAGAGAAGATTAGCATGGCC |
| U6-R | CGATTTTGCGTGTCATCCTT |
